# Das mykotische Aortenaneurysma – repräsentative Fälle: Fallserie von klinischen Fallbeispielen und die aktuelle Studienlage zu deren Therapieoptionen der offen-chirurgischen Resektion mit Prothesenimplantation (OR) sowie endovaskulärem Aortenrepair (EVAR)

**DOI:** 10.1007/s00104-025-02375-z

**Published:** 2025-09-16

**Authors:** E. Schwarz, P. Arndt, F. Meyer, M. Pech, U. Barth

**Affiliations:** 1 Arbeitsbereich Gefäßchirurgie, Klinik für Allgemein‑, Viszeral‑, Gefäß- und Transplantationschirurgie, Universitätsklinikum Magdeburg A.ö.R., Leipziger Str. 44, 39120 Magdeburg, Deutschland; 2 Klinik für Allgemein‑, Viszeral‑, Gefäß- und Transplantationschirurgie, Universitätsklinikum Magdeburg A.ö.R., Magdeburg, Deutschland; 3 Klinik für Radiologie und Nuklearmedizin, Universitätsklinikum Magdeburg A.ö.R., Magdeburg, Deutschland

**Keywords:** Aortenaneurysmaspektrum, Offen-chirurgische Rekonstruktion (OR), Endovaskuläres Repair (EVAR), Case report/series, Review, Aortic aneurysm spectrum, Open surgical repair (OR), Endovascular repair (EVAR), Case report/series, Review

## Abstract

**Hintergrund:**

Das mykotische Aortenaneurysma (MAA) ist eine seltene lebensbedrohliche Erkrankung. Therapeutisch steht die offen-chirurgische Resektion (OR) mit Prothesenimplantation dem endovaskulären Aortenrepair (EVAR) gegenüber.

**Ziel:**

Darstellung der Therapieentscheidung anhand von Fallbeispielen mit Diskussion der aktuellen Studienlage.

**Fälle:**

1. Bei der notfallmäßigen Resektion eines gedeckt rupturierten Bauchaortenaneurysmas (BAA) einer 74-jährigen Patientin zeigt sich ein MAA. Es wird ein Xenograft implantiert. Im Verlauf kommt es durch Komorbiditäten zum Exitus letalis. 2. Bei einem 79-jährigen Patienten mit MAA als Fokus einer Salmonellensepsis erfolgt ein EVAR mit Stentgraft. Eine gute Prothesenlage und Perfusion werden festgestellt. Am 7. postoperativen Tag wird der Patient mit Langzeitantibiotikatherapie entlassen. 3. Ein 63-jähriger Patient mit gedeckt rupturiertem MAA und Psoasabszess wird notfallmäßig mittels OR und Implantation einer Xenograft-Prothese versorgt. Am 12. postoperativen Tag wird der Patient mit Langzeitantibiotikatherapie entlassen.

**Diskussion:**

Die Analyse von 21 Studien (2433 Patienten) zeigte bessere Kurzzeitergebnisse bei EVAR (3-Monats-Überleben 96 % *vs*. 74 % bei OR), aber höhere Reinfektionsraten (42 % *vs*. 18 %). Das 5‑Jahres-Überleben ist ähnlich (EVAR: 57–79,7 %; OR: 60 %). Häufigste Erreger waren Salmonellen (26,3 %) sowie *Staphylococcus aureus* (13,9 %). Blutkulturen blieben bei 37,4 % negativ. Risikofaktoren sind ein fortgeschrittenes Alter, männliches Geschlecht und Komorbiditäten. Die Antibiotikatherapie von 6 Monaten verbessert das Outcome.

**Fazit:**

Die Wahl der Therapie erfolgt individuell. Beide Verfahren zeigen ähnliche Langzeitergebnisse bei unterschiedlichen Frühkomplikationen. Die langfristige Antibiotikatherapie ist essenziell für ein optimales Outcome.

**Graphic abstract:**

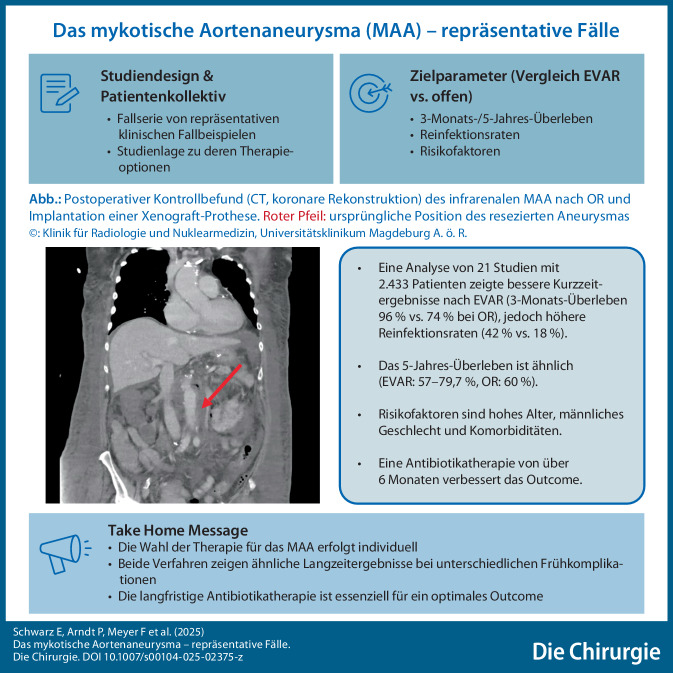

## Abstract (en)

**Background:**

The mycotic aortic aneurysm (MAA) is a rare life-threatening condition. Treatment options include open surgical resection (OR) with prosthesis implantation and endovascular aortic repair (EVAR).

**Aim:**

Presentation of the therapeutic decisions illustrated by clinical case examples and discussion of current references from the medical scientific literature.

**Case reports:**

1. An emergency resection of a covered ruptured abdominal aortic aneurysm (AAA) in a 74-year-old female patient revealed a MAA. A xenograft was implanted. Ultimately, the comorbidities led to death. 2. A 79-year-old male patient with a MAA as the focus of a *Salmonella* sepsis underwent EVAR using implantation of a stent graft. Correct graft placement and adequate perfusion were confirmed. On postoperative day 7, the patient was discharged with long-term antibiotic therapy. 3. A 63-year-old male patient with a covered ruptured AAA and psoas muscle abscess underwent emergency surgery with OR and implantation of a xenograft prosthesis. On postoperative day 12 the patient was discharged with long-term antibiotic therapy.

**Discussion:**

An analysis of 21 studies (2433 patients) showed a better short-term survival using EVAR (3-month survival, 96% *vs*. 74% for OR) but a higher reinfection rate (42 % *vs*. 18 %). The 5‑year survival rates are similar (EVAR: 57–79.7%, OR: 60%). The most common pathogens were *Salmonella* (26.3%) and *Staphylococcus aureus* (13.9%). Blood cultures remained negative in 37.4%. Risk factors are advanced age, male gender and comorbidities. A long-term antibiotic therapy of > 6 months improves the outcome.

**Conclusion:**

The treatment of MAA requires an individualized approach as both OR and EVAR exhibit similar long-term outcomes but different early complication rates. Long-term antibiotic therapy is essential for an optimal outcome.

## Einleitung

Das mykotische (Bauch‑)Aortenaneurysma (M[B]AA) ist ein seltenes Krankheitsbild mit einer hohen Mortalität. Im Anschluss an die Bestätigung der Diagnose stellt sich (stets) die Frage, ob eine Therapie mittels offen-gefäßchirurgischer Resektion (OR) und Prothesenimplantation oder mittels endovaskulären Aortenrepairs (EVAR) indiziert ist [1].

*Ziel* des vorliegenden Manuskriptes war es, basierend auf

i eigenen gewonnenen Fall- und Diagnose-spezifischen Managementerfahrungen (sowie)
ii einschlägigen, themenbezogenen Referenzen der aktuellen medizinisch-wissenschaftlichen Literatur,

einen Überblick über die Kriterien für die Therapieentscheidung zwischen OR und EVAR zu erstellen und damit dem Kliniker eine Entscheidungshilfe im perioperativen Management aus derzeit verfügbarer Datenansicht an die Hand zu geben.

## Methode

Es werden 3 Patienten beschrieben, welche durch den berichtenden Arbeitsbereich Gefäßchirurgie zwischen November 2024 und Februar 2025 behandelt wurden, um Spezifika des befundgerechten diagnostischen und therapeutischen Managements des MBAA aufzuzeigen.

Zur anschließenden Diskussion wurde die Studienlage anhand der aktuell verfügbaren Referenzen aus der themenbezogenen medizinisch-wissenschaftlichen Literatur analysiert und mit Fokus auf der Therapieentscheidung sowie deren jeweiligen Vor- und Nachteilen ausgewertet. Dafür wurde eine englischsprachige Suche mittels PubMed^®^ nach den Suchwörtern „aortic“, „aneurysm“, „EVAR“, und „open surgery“ durchgeführt. Ausschlusskriterien waren solche Treffer, die ausschließlich zu einem Abstract ohne Volltext führten, sowie Fragestellungen, deren thematischer Schwerpunkt über den Kernbereich dieser Arbeit hinausging. Die Auswertung der Literatur erfolgte basierend auf der Zusammenfassung von Studienergebnissen, deskriptiver Statistik und relativer Häufigkeiten.

### Stellungnahme

Die Studie wurde gemäß den Richtlinien der „Deklaration von Helsinki für die biomedizinische Forschung“ aus dem Jahr 1964 und deren weiteren Änderungen, entsprechend den Anweisungen der institutionellen Ethikkommission sowie gemäß den Regeln der „Guten Klinischen Praxis und Forschung“ durchgeführt.

Jeder Patient unterzeichnete vor dem chirurgischen Eingriff eine Einverständniserklärung, nachdem der verantwortliche Chirurg den Patienten in einem ausführlichen persönlichen Gespräch über die einzelnen Schritte und möglichen Komplikationen des chirurgischen Eingriffs aufgeklärt und alle Fragen und Bedenken des Patienten verständlich beantwortet hatte.

Die Daten enthalten personenbezogene Informationen gemäß dem Datenschutzgesetz und können aus ethischen Gründen nicht ohne entsprechende Genehmigungen öffentlich zugänglich gemacht werden. Entsprechend § 17 Abs. 1 S. 2 des Krankenhausgesetzes des Landes Sachsen-Anhalt wurden die im Rahmen der Krankenhausbehandlung erhobenen und gespeicherten Patientendaten vor ihrer weiteren Verarbeitung anonymisiert und konnten dadurch auch ohne Einwilligung bei berechtigtem Interesse der Allgemeinheit an der Durchführung des Forschungsvorhabens verwendet werden.

## Ergebnisse (Mini-Fallserie)

### Fall 1

#### Anamnese

Eine 74-jährige Patientin stellte sich aufgrund von Fieber, Abgeschlagenheit, Müdigkeit, Kraftlosigkeit mit Luftnot sowie zunehmenden Brust- und Bauchschmerzen in der Notaufnahme vor.

Eigenanamnestisch ließ sich eine vorbestehende chronische Niereninsuffizienz Grad IIa eruieren, weshalb als Arbeitsdiagnosen ein akutes Nierenversagen sowie ein BAA in Betracht gezogen wurden. Trotz des Verdachtes auf ein BAA wurde die Patientin zunächst nur internistisch gesehen. Bei abfallendem Hämoglobin(Hb)-Wert und zunehmender Verschlechterung des Allgemeinzustandes (Az) der Patientin wurde diese zur dringlich angezeigten bildgebenden Diagnostik bei anzunehmendem, dringend drohendem hämorrhagischen Schockgeschehen stationär aufgenommen.

#### Klinischer Befund

Bis auf die Verschlechterung des Az präsentierte sich die Patientin als klinisch wenig auffällig.

#### Diagnostik

##### Labor*.*

Während der Diagnostik zeigte sich bei der Patientin ein stetiger Hb-Abfall von 5,4 auf 3,9 mmol/l innerhalb von 23 h, was das vermutete Schockgeschehen bestätigte. Zum Zeitpunkt der Aufnahme lag das CRP bei 246,0 mg/l. Die Patientin wies eine glomeruläre Filtrationsrate (GFR) von 32,03 ml/min auf. Zusätzlich wurden eine Anisozytose, Poikilozytose sowie eine Alkalose (pH: 7,525) festgestellt.

Die abgenommenen Blutkulturen waren positiv auf *Aeromonas veronii*.

##### Bildgebung*.*

In einer Röntgenaufnahme des Thorax vom Aufnahmetag zeigte sich ein deutlicher Zwerchfellhochstand links.

In einer aufgrund des raschen Hb-Abfalls durchgeführten Abdomensonographie zeigte sich neben polyzystischen Degenerationen beider Nieren eine infrarenale Dilatation der Aorta abdominalis von etwa 6 cm im Durchmesser mit schalenförmiger Thrombusbildung.

**Abb. 1:**
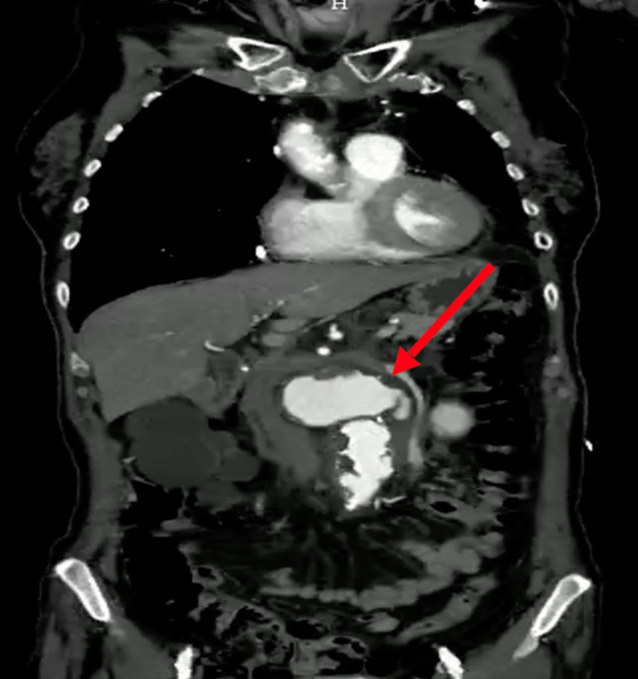
Ausgangsbefund des infrarenalen BAA mit retroperitonealer Perforation – CT-Aufnahme des Abdomens (koronare Rekonstruktion; Fall 1). Der *rote Pfeil* markiert das BAA (Quelle: Klinik für Radiologie und Nuklearmedizin, Universitätsklinikum Magdeburg A. ö. R.)

Zur genaueren Beurteilung und definitiven Diagnosestellung wurde im Anschluss daran eine Computertomographie (CT) des Abdomens durchgeführt (Abb. 1). Hierbei konnte die infrarenale Aortendilatation mit einer maximalen Größe von 6,8 × 5,3 cm und einer kraniokaudalen Ausdehnung von etwa 5,1 cm des erweiterten Aortendurchmessers bestätigt werden. Die Aortenwand im Dilatationsbereich zeigte eine unscharfe Begrenzung. Retroperitoneal wurde eine Flüssigkeitsansammlung festgestellt. Beide Beschreibungen können als hinweisgebendes, aber unsicheres Zeichen für ein infektiöses Geschehen gewertet werden.

*Gedeckt rupturiertes infrarenales Bauchaortenaneurysma (5,8* *×* *6,3* *cm) mit Perforation nach retroperitoneal.*

#### Therapie

Bei der Diagnose eines gedeckt rupturierten BAA wurde nach Vorstellung unmittelbar die Indikation für eine notfallmäßige OR des Aneurysmas durch die Gefäßchirurgie gestellt.

##### Operativ.

**Abb. 2:**
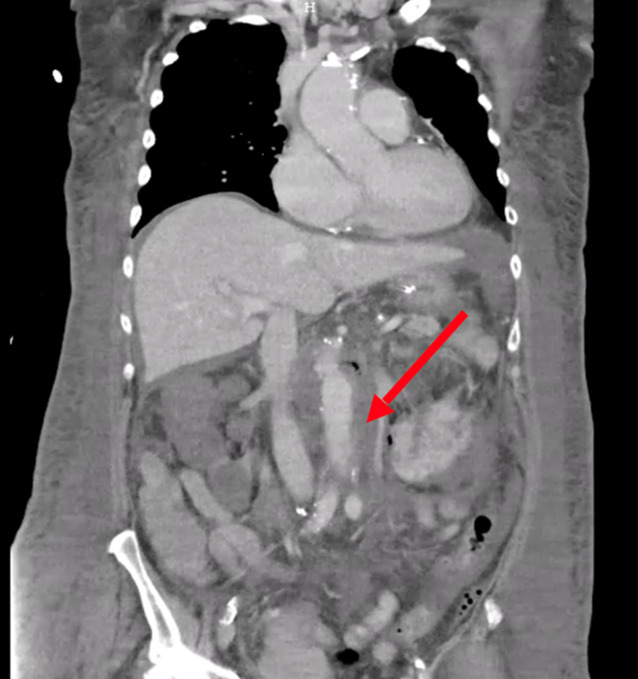
Postoperativer Kontrollbefund (CT, koronare Rekonstruktion) des infrarenalen MBAA nach OR und Implantation einer Xenograft-Prothese – Fall 1. Der *rote Pfeil* markiert die ursprüngliche Position des resezierten Aneurysmas (Quelle: Klinik für Radiologie und Nuklearmedizin, Universitätsklinikum Magdeburg A. ö. R.)

Intraoperativ zeigte sich die Aneurysmawand als stark entzündlich verändert. Es wurde der dringende Verdacht auf ein MAA erhoben. Eine Probe der Aortenwand wurde zur mikrobiologischen Aufarbeitung gesichert. Die Resektion des Aneurysmas verlief erfolgreich (postoperative CT-Kontrolle: Abb. 2). Aufgrund des Entzündungsgeschehens traf das OP-Team intraoperativ die Entscheidung, eine Xenograft-Prothese aus Rinderperikard (20 × 10 × 320 mm BioIntegral Surgical No-React^®^ bovine pericardial xenograft; BioIntegral Surgical Inc., Mississauga/ON, Canada) zu implantieren, da Xenografts trotz einer geringeren langfristigen Offenheitswahrscheinlichkeit im Vergleich zu Allografts aus Kunststoff als deutlich infektresistenter gelten.

Postoperativ wurde die Patientin auf die Intensivstation verlegt.

##### Systemisch.

Nach mikrobiologisch determinierter Erweiterung der Diagnose auf ein MBAA mit gedeckter Ruptur bei retroperitonealer Perforation wurde die bisherige Breitspektrumantibiotikatherapie mit 4‑mal 4 g Piperacillin und 0,5 g Tazobactam i. v. um eine dem Resistogramm adaptierte Gabe von Ciprofloxacin 400 mg i. v. 2‑mal täglich erweitert.

#### Histopathologie

In der histopathologischen Aufarbeitung der Aneurysmawand konnte ein hochflorides entzündliches Geschehen festgestellt werden, welches kausal an der Ruptur des Aneurysmas beteiligt gewesen sein könnte.

#### Verlauf

Aufgrund des hämorrhagischen Schocks nach Aneurysmaruptur kam es zu einem dialysepflichtigen Nierenversagen der Patientin. Bei neu aufgetretenen Teerstühlen in Verbindung mit einem weiterbestehenden Hb-Abfall (von 5,7 auf 4,9 mmol/l) wurde eine Koloskopie durchgeführt. Hierbei zeigte sich eine Neoplasie im Bereich der rechten Kolonflexur; die entnommenen Probebiopsien ergaben ein Karzinom.

Aufgrund eines prolongierten Weanings wurde eine Tracheotomie vorgenommen.

Im Gespräch mit den Angehörigen wurde letztlich nach mutmaßlichem Willen der zu diesem Zeitpunkt nicht aufklärungsfähigen Patientin die Entscheidung getroffen, die Therapiemaßnahmen nicht weiter zu eskalieren im Sinne eines „best supportive care“.

Vier Wochen postoperativ kam es zum *Exitus letalis naturalis *aufgrund eines akuten Nierenversagens nach Abbruch der Dialysetherapie.

### Fall 2

#### Anamnese

Ein 79-jähriger Patient stellte sich in der Notaufnahme aufgrund einer erneuten Verschlechterung seines Az, Fieber und Schüttelfrost bei Zustand nach *Salmonellen*-Sepsis vor 3 Monaten vor. Nach initial erfolgreicher antibiotischer Sanierung waren die Blutkulturen nun erneut positiv für *Salmonellen*. Zusätzlich berichtete der Patient über neu aufgetretene stechende Rückenschmerzen. Zur Fokussuche und Therapie wurde der Patient stationär aufgenommen.

#### Klinischer Befund

Der Patient präsentierte sich mit reduziertem Az. Die körperliche Untersuchung war unauffällig. Das Atemgeräusch war vesikulär ohne Nebengeräusche, das Abdomen weich und ohne Druckschmerz.

#### Diagnostik

##### Labor*.*

Über den gesamten Krankheitsverlauf zeigten sich die Leukozyten im Normbereich. Es kam zu einem leichten Hb-Abfall auf 7,6 mmol/l, welcher im Verlauf stabil blieb. Weiterhin kam es zu einem CRP-Anstieg von 32,70 auf 136 mg/l. Abgenommene Blutkulturen waren positiv auf *Salmonella enteritidis*.

##### Bildgebung*.*

Bei einliegender Hüftgelenksprothese (HG-Totalendoprothese [TEP] links 2021) erfolgte zunächst eine Fokussuche mittels Röntgen des Beckens in 2 Ebenen. Eine Prothesenlockerung konnte so ausgeschlossen werden. Eine CT des Thorax und Abdomens zeigte einen pathologisch vergrößerten retroaortalen Lymphknoten rechts auf Höhe des BWK12 sowie ein suprazöliakales penetrierendes Aortenulkus (PAU) mit leicht unscharfer Begrenzung auf Höhe des LWK 1.

**Abb. 3:**
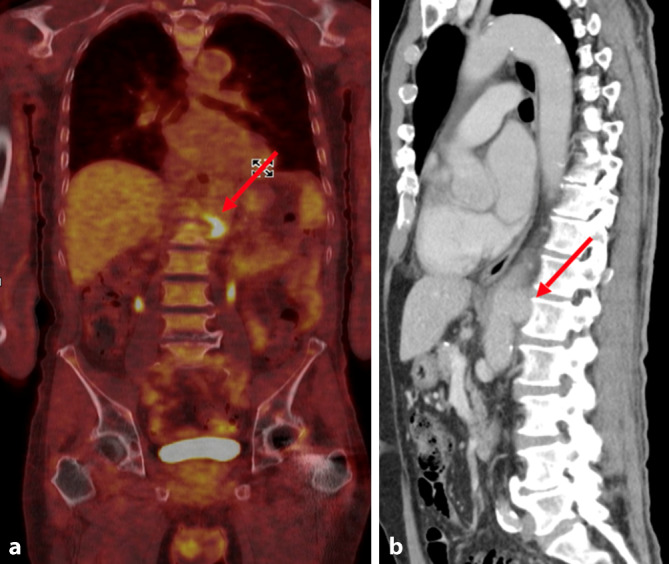
Ausgangsbefund des symptomatischen suprazöliakalen mykotischen penetrierenden Aortenulkus (PAU) – Fall 2. **a** „^18^F‑fluoro-D-deoxyglucose positron emission tomography“ (PET)/CT mit fokal erhöhtem Glukosestoffwechsel im Bereich eines Aneurysmas der Aorta abdominalis. **b** Computertomographische Aufnahme des PAUs (sagittale Rekonstruktion). Die *roten Pfeile* zeigen auf das Aortenaneurysma (Quelle: Klinik für Radiologie und Nuklearmedizin, Universitätsklinikum Magdeburg A. ö. R.)

Schließlich ließ sich in einem FDG-PET-CT („^18^F‑fluoro-D-deoxyglucose positron emission tomography“/CT) ein fokal erhöhter Glukosestoffwechsel im Bereich thrombotischer Wandauflagerungen einer dilatierten Aorta abdominalis auf Höhe des LWK 1 als wahrscheinlicher persistierender Infektfokus identifizieren (Abb. 3).

Symptomatisches suprazöliakales mykotisches penetrierendes Aortenulkus (PAU) mit Nachweis von Salmonella enteritidis in der Blutkultur.

#### Therapie

In der interdisziplinären Gefäßkonferenz wurde gemeinsam mit den Kollegen der Radiologie die Indikation für eine interventionelle Therapie des PAU gestellt. Aufgrund der Lage des PAUs sowie des Alters und Az des Patienten wurde die Entscheidung zur Versorgung mittels EVAR getroffen.

##### Operativ/interventionell*.*

Zunächst wurde die linke A. femoralis communis punktiert und eine 5‑F-Schleuse (5 French, 65 cm, Tip Shape C2, Guidewire Compatibility 0,038, Imager™ II; Fa. Boston Scientific, Marlborough, MA, USA) eingeführt. Mittels 0,035″-Draht (5 French, 80 cm, 0,038″ NB, Soft-Vu Angiographic Katheter; Fa. Angiodynamics, Latham, NY, USA) erfolgte die Sondierung der A. mesenterica superior. Durch die chirurgisch freigelegte rechte Leiste wurde eine 11-F-Schleuse (11 French, 50 cm, Einführschleuse mit Hämostaseventil; Fa. Bisping Medizintechnik GmbH, Aachen, Deutschland) eingeführt und der Truncus coeliacus mittels 0,035″-Draht (5 French, 80 cm, 0,038″ NB, Soft-Vu Angiographic Katheter; Fa. Angiodynamics, Latham, NY, USA) sondiert. Nach temporärer Blockierung des proximalen Truncus coeliacus mittels Ballonkatheter (8 × 20 mm, Abbott Vascular Armada; Fa. Abbott, Abbott Park, IL, USA) und Kontrastmittelgabe über den in der A. mesenterica superior liegenden Katheter wurde der retrograde Fluss über die A. gastroduodenalis gesichert.

**Abb. 4:**
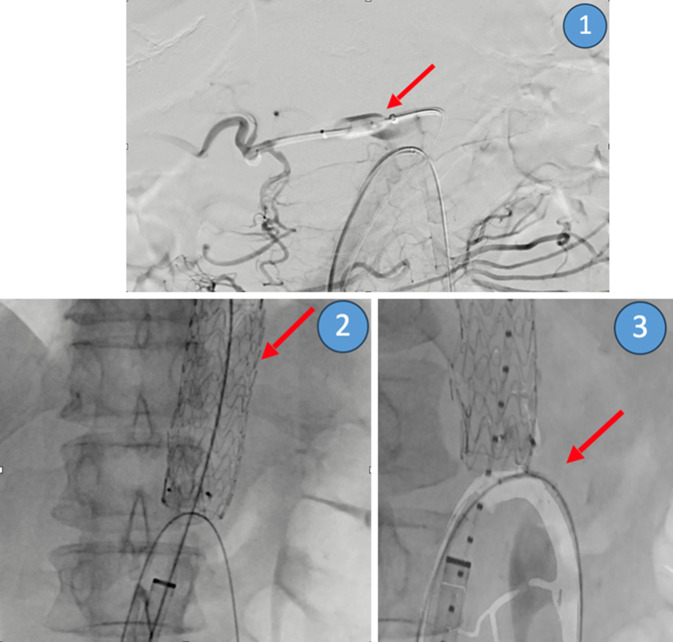
Intraoperativer Befund der Versorgung des mykotischen PAUs (Fall 2) mittels EVAR: (*1*) Testokklusion des Truncus coeliacus mittels Plugembolisation (*roter Pfeil*), um den retrograden Fluss der A. gastroduodenalis zu beweisen; (*2*) EVAR mittels Thoracic-Stentgraft-Prothese (*roter Pfeil*); (*3*) Stentimplantation in die A. mesenterica superior (*roter Pfeil*), um ein Abrutschen der Graft-Prothese über die A. mesenterica superior zu verhindern (Quelle: Klinik für Radiologie und Nuklearmedizin, Universitätsklinikum Magdeburg A. ö. R.)

Anschließend wurde der proximale Truncus coeliacus durch eine Plugembolisation (10 mm Amplatzer Vascular Plug II; Fa. Abbott, Abbott Park, IL, USA) verschlossen. Zur Überprüfung der retrograden Perfusion der A. gastrica sinistra wurde eine Kontrollserie angefertigt (Abb. 4).

Zur Versorgung des bildmorphologisch infektiösen PAUs der distalen Aorta thoracica erfolgten eine Sondierung des echten Lumens mit einem 0,035″ Lunderquist-Draht (0,035 inch, 11 cm Taper Lenght, Lunderquist™ Extra Stiff Wire Guide; Fa. Cook Medical, Bloomington, IN, USA) und die Einbringung eines Stentgrafts in die distale thorakale Aorta. Es erfolgten eine komplikationslose Stentimplantation einer Thoracic-Stentgraft-Prothese (10 cm × 31 mm Gore TAG Conformable Thoracic-Stentgraft-Prothese; W. L. Gore & Associates, Putzbrunn, Deutschland) sowie eine Stentimplantation der proximalen A. mesenterica superior (8 mm × 20 mm × 130 cm, LifeStent Vascular Stent System; Fa. BD Switzerland, Eysins, Schweiz) mit Kontrollserien (Abb. 4).

**Abb. 5:**
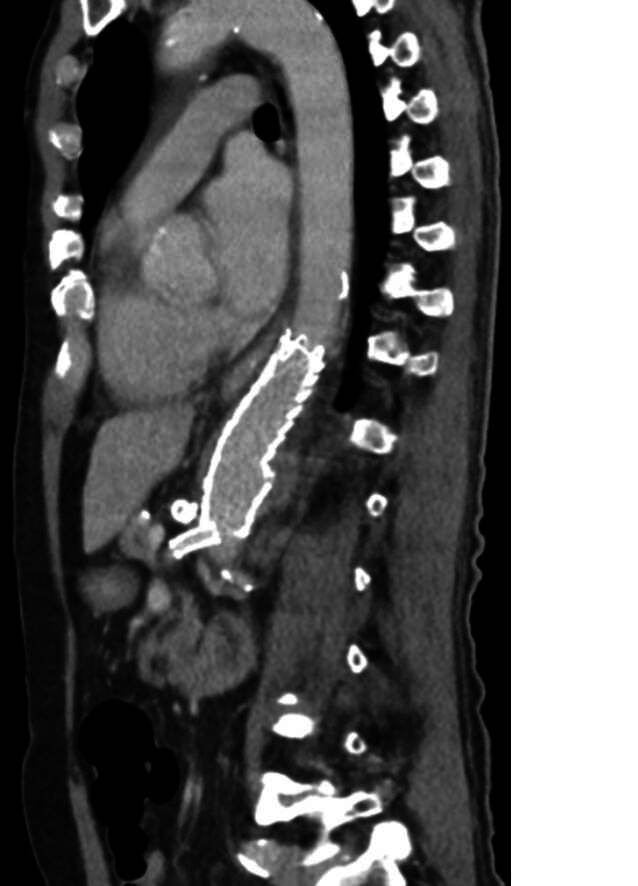
Postoperativer Befund nach Versorgung des mykotischen PAUs – Fall 2 – mittels EVAR (Quelle: Klinik für Radiologie und Nuklearmedizin, Universitätsklinikum Magdeburg A. ö. R.)

Eine abschließende postoperative CT-Kontrolle bestätigte den anhaltenden Erfolg der Resektion mit regelrechter Kontrastierung der implantierten Xenograft-Prothese, eine Kontrastmittelanreicherung im Bereich des entlasteten Psoasabszesses, bei der es sich am ehesten um Granulationsgewebe handelte, sowie neu aufgetretene Pleuraergüsse beidseits (Abb. 5). Im Untersuchungsgebiet war keine abszessverdächtige Formation zu detektieren.

##### Systemisch*.*

Aufgrund der positiven Blutkulturen wurde die kalkulierte Antibiotikatherapie mit Ciprofloxacin 2-mal 400 mg i. v. Resistogramm-gerecht fortgeführt.

#### Verlauf

Die Symptomatik des Patienten zeigte sich postoperativ rückläufig. Aufgrund einer Schwellung im Bereich des Zugangs zur Stentprothese in der rechten Leiste wurde im weiteren Verlauf mittels Ultraschall ein Hämatom von etwa 3 × 2 cm Größe festgestellt. Nach entsprechender Aufklärung und Vorbereitung des Patienten erfolgten die operative Wundrevision, Ausräumung und Spülung des Hämatoms. Der weitere Verlauf auf der gefäßchirurgischen Normalstation gestaltete sich unauffällig.

Nach Rücksprache mit den Kollegen der Gastroenterologie und Infektiologie wurden eine Fortsetzung der Antibiotikagabe mittels Cotrimoxazol oral 960 mg 2‑mal täglich p. o. für weitere 6 Monate und anschließende laborchemische Erfolgskontrolle mittels Blutkulturen sowie Anbindung an die einrichtungsinterne Infektiologieambulanz empfohlen.

Der Patient konnte in gutem Az mit reizlosen Wundverhältnissen am 8. postoperativen Tag nach Hause entlassen werden. Eine Verlaufskontrolle nach 6 Wochen ergab ein CRP von 4 (SI) sowie weiterhin negative Blutkulturen.

### Fall 3

#### Anamnese

Ein 63-jähriger Patient stellte sich in der zentralen Notaufnahme mit einer Überweisung durch seinen Hausarzt vor. Bei seit 4 Wochen bestehenden Rückenschmerzen war durch den Hausarzt eine CT-Untersuchung des Abdomens veranlasst worden, welche ein gedeckt rupturiertes BAA nach links-lateral bei Verdacht auf einen Psoasabszess sowie eine Spondylodiscitis zeigte. Der Patient stellte sich daraufhin mit einer Überweisung in der hiesigen zentralen Notaufnahme vor und wurde zur notfallmäßigen Versorgung stationär aufgenommen.

#### Klinischer Befund

Der Patient wies anhaltende Rückenschmerzen und Bauchschmerzen bei mäßiger Palpation („leichter Druck“) auf. Atmung und Kreislauf waren stabil.

#### Diagnostik

##### Labor (SI).

Der Patient wies zum Aufnahmezeitpunkt einen Hb-Wert von 8,80 mmol/l auf. Das CRP lag bei 35,9 mg/l.

##### Bildgebung*.*

**Abb. 6:**
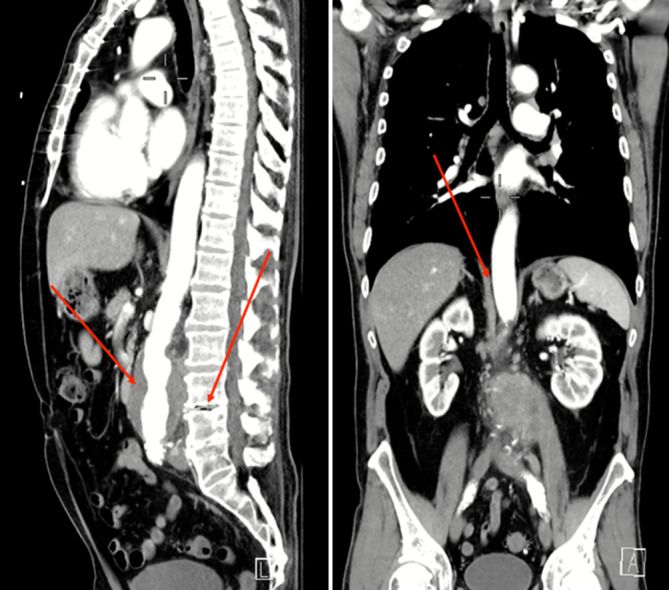
Ausgangsbefund (CT, sagittale und koronare Rekonstruktionen) des infrarenalen MBAA und angrenzender entzündlicher Prozesse im M. psoas links sowie dorsalseitig im Bandscheibenfach L3/L4. Die *roten Pfeile* zeigen auf das Aortenaneurysma, Lufteinschlüsse im Bandscheibenfach L3/L4 und den Psoasabszess (Quelle: Klinik für Radiologie und Nuklearmedizin, Universitätsklinikum Magdeburg A. ö. R.)

Da der Patient den zuvor erstellten CT-Befund nicht zur Reevaluation mitbrachte, wurde in der Notaufnahme zur Diagnosesicherung und Therapieentscheidung ein CT des Abdomens aus Dringlichkeitsgründen durchgeführt (Abb. 6). Hierbei zeigte sich ein infrarenales Aortenaneurysma mit einem maximalen Durchmesser von 5,5 cm und einem angrenzenden am ehesten entzündlichen Prozess von 26 × 36 mm im M. psoas links sowie dorsalseitig im Bandscheibenfach L3/L4. Das Vorhandensein von Lufteinschlüssen in diesen Bereichen ergab den dringenden Verdacht auf ein gedeckt rupturiertes Bauchaortenaneurysma mit Psoasabszess und Spondylodiscitis.

Gedeckt rupturiertes infrarenales Bauchaortenaneurysma (5,8 *×* *6,3* cm) mit Psoasabszess links sowie Spondylodiszitis.

#### Therapie

Aufgrund der Diagnose eines gedeckt rupturierten BAAs mit Psoasabszess wurde die Indikation für eine notfallmäßige OR des Aneurysmas und Ausräumung des Psoasabszesses gestellt.

##### Operativ*.*

Da bereits präoperativ ein starker Verdacht auf ein mykotisches BAA gestellt worden war, wurde zur Vorbereitung der notfallmäßigen Versorgung mittels OR eine Rohrprothese aus einer Perikardpatchplatte (8 × 14 cm, Peri-Guard^®^ Repair-Patch; Lamed, Oberhaching, Deutschland) mittels fortlaufender Naht (5‑0, 45 cm, Prolene, blau monofil; Fa. Ethicon Johnson & Johnson, Norderstedt, Deutschland) zweireihig genäht.

Zur Blockung und Sicherung der Perforationsstelle des BAAs wurde ein Ballonkatheter (8 F, 10–46 mm × 100 cm; Fa. Medtronic, Minneapolis, MN, USA) bis suprarenal vorgeschoben und dilatiert. Die Bauchhöhle wurde in typischer Art eröffnet und das Aneurysma dargestellt. Das umgebende Gewebe zeigte sich entzündlich verändert. Während der Resektion des Aneurysmas wurde eine dorsale Perforationsstelle gefunden, die nach links-lateral in einen entzündlichen Prozess des M. psoas mündete. Für die mikrobiologische Aufbereitung wurden Proben aus der Aneurysmawand sowie dem M. psoas entnommen. Die weitere Resektion des Aneurysmas und Implantation der zuerst vorbereiteten Xenograft-Rohrprothese aus Rinderperikard (8 × 14 cm, Peri-Guard^®^ Repair-Patch; Lamed, Oberhaching, Deutschland) verlief erfolgreich. Der Patient wurde zur postoperativen Überwachung zunächst auf die Intensivstation verlegt und konnte am 2. postoperativen Tag auf die gefäßchirurgische Normalstation übernommen werden.

##### Systemisch*.*

Bereits nach Sichtung der CT-Aufnahmen in der Notaufnahme wurde eine antibiotische Therapie mittels je 4‑mal 4 g Piperacillin und 0,5 g Tazobactam i. v. begonnen. Im Anschluss an die Operation wurde diese nach Resistogramm adaptiert und um 3‑mal 200 mg Erythromycin i. v. erweitert.

#### Mikrobiologie

Eine mikrobiologische Aufarbeitung der intraoperativ entnommenen Proben ergab eine kulturelle Besiedlung durch Bakterien der *Clostridium* spp. (eine genaue Identifizierung ergab *Robinsoniella peoriensis*) sowie *Staphylococcus epidermidis* und *Staphylococcus aureus*.

#### Histopathologie

Es wurde ein hochflorides entzündliches Geschehen festgestellt, welches kausal an der Ruptur des Aneurysmas beteiligt gewesen sein könnte.

#### Verlauf

Postoperativ kam es zu einem starken Anstieg der Entzündungsparameter mit einem CRP von präoperativ 30,5 mg/l auf 198,0 mg/l am 4. postoperativen Tag. Es kam weiterhin zu einem Hb-Abfall von präoperativ 8,80 auf 5,20 mmol/l am 4. postoperativen Tag. Im weiteren Verlauf stabilisierten sich die Werte mit einem CRP von 34,1 mg/l und einem Hb von 5,6 mmol/l am 10. postoperativen Tag.

**Abb. 7:**
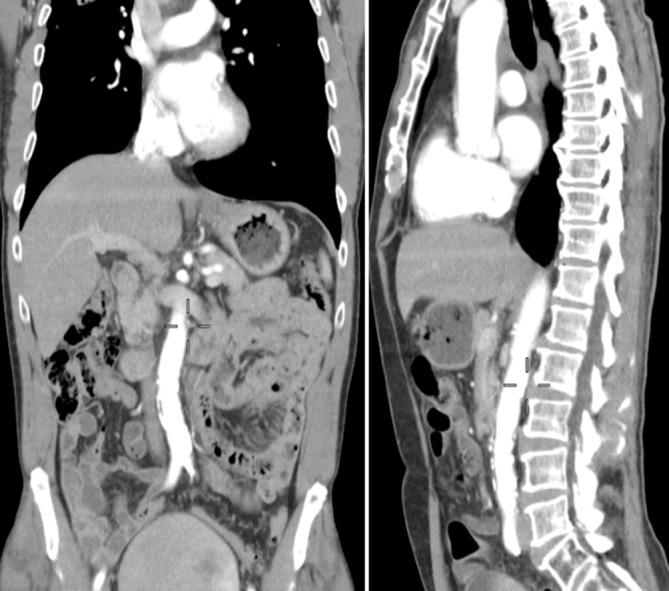
Postoperativer Befund nach Versorgung des MAA mit regelrechter Kontrastierung der eingelegten Patch-Prothese und Sanierung des Psoasabszesses (Quelle: Klinik für Radiologie und Nuklearmedizin, Universitätsklinikum Magdeburg A. ö. R.)

Eine abschließende postoperative CT-Kontrolle (Abb. 7) bestätigte den anhaltenden Erfolg der Resektion mit regelrechter Kontrastierung der implantierten Xenograft-Prothese, eine Kontrastmittelanreicherung im Bereich des entlasteten Psoasabszesses, bei der es sich am ehesten um Granulationsgewebe handelte, sowie neu aufgetretene Pleuraergüsse beidseits.

In Rücksprache mit den Kollegen der Infektiologie wurde die Empfehlung einer Fortführung der Antibiotikagabe mittels Clindamycin oral 600 mg 1‑mal täglich für weitere 6 Monate und regelmäßiger laborchemischer Verlaufskontrollen mittels Blutkulturen sowie Anbindung an die einrichtungsinterne Infektiologieambulanz gestellt.

Am 12. postoperativen Tag konnte der Patient in gutem Az und mit reizlosen Wundverhältnissen entlassen werden.

## Diskussion

### Definition

Der Begriff des MBAA wird definiert als jedes BAA mit infektiöser Komponente. Es ist dabei nicht immer zweifelsfrei zu klären, ob Patienten primär ein BAA aufwiesen, in dessen Bereich sich Erreger angesiedelt haben, oder ob durch eine mikrobielle Kolonisierung eines Bereiches der Bauchaorta sich eine Schädigung der Aortenwand manifestiert hat mit sich anschließender Generierung eines BAA. Grundsätzlich lässt sich feststellen, dass gesunde Gefäße als relativ infektresistent gelten. Bei den meisten Patienten dürfte daher eine vorbestehende Schädigung der Bauchaorta als Hauptrisikofaktor für ihre bakterielle Besiedlung infrage kommen. Weitere Risikofaktoren für das Entstehen eines MBAAs stellen Drogenabusus, Traumata, Immunsuppression sowie einliegende Gefäßprothesen dar. Zu den häufigsten Erregern gehören Staphylokokken, Salmonellen und *E. coli*. Sehr selten kommen auch Mischinfektionen gramnegativer Keime oder Pilzinfektionen vor. In Asien lässt sich außerdem eine Häufung von Infektionen durch Salmonellen im Vergleich zu westlichen Ländern erkennen. Grund dafür könnte die Ernährung mit einem höheren Anteil an rohem oder selbst geschlachtetem Fleisch sein. Typische Symptome gibt es für das MBAA praktisch nicht. Mit eher unspezifischen Symptomen wie Fieber, Schmerzen und Verschlechterung des Allgemeinzustandes wird die korrekte Diagnose häufig erst in fortgeschrittenen Stadien der Erkrankung gestellt [2].

Für eine umfassende Analyse der entsprechenden Literatur wurde die medizinische Datenbank Medline mittels englischsprachigen Schlagworten wie „mycotic“, „aortic“, „aneurysm“, „EVAR“, und „open surgery“ durchsucht. Diese Suche ergab 38 Treffer. Nach Ausschluss von Treffern, die ausschließlich zu einem Abstract ohne Volltext führten, sowie Ausschluss von Publikationen und Fragestellungen, deren thematischer Schwerpunkt über den Kernbereich dieser Arbeit hinausging, blieben 21 Publikationen [3–22]. Die Auswertung der Daten dieser Publikationen in Form einer deskriptiven Statistik mit relativen Häufigkeiten erfolgte unter Verwendung des Programms IBM^®^ SPSS^®^ Statistics Version 29.0.2.0 (Chicago, IL, USA).

Insgesamt wurden so 2433 Patienten mit mykotischem Bauchaortenaneurysma in die Aufarbeitung eingeschlossen.

Von diesen Patienten wurden 1291 (53,1 %) mittels OR, 1108 (45,5 %) durch EVAR und 34 (1,4 %) konservativ therapiert. Die beschriebenen Patienten waren zum überwiegenden Teil Männer (79,0 %) mit einem Durchschnittsalter von 69,31 ± 1,59 Jahren. Bekannte kardiovaskuläre Vorerkrankungen wiesen 78,6 % der Patienten auf. Eine Immunsuppression lag bei 20,1 % vor.

### Diagnostik

Diagnostisch kommen meist 2 Szenarien infrage: Entweder wurde das BAA festgestellt und so wie in dem oben beschriebenen Fall erst später bzw. intraoperativ eine Entzündung dessen (mit dem entwickelten dringenden Verdacht auf MBAA) bemerkt, oder bei Patienten wird eine Bakteriämie festgestellt, und im Rahmen der Fokussuche zeigt sich ein bisher unbekanntes BAA.

Labordiagnostisch sind Entzündungsparameter (C-reaktives Protein, Procalcitonin) sowie positive Blutkulturen wegweisend. Für die endgültige Diagnose ist jedoch die CT-Angiographie (computertomographische Angiographie unter Verwendung von Kontrastmittel) das Mittel der Wahl [2]. Zu den Zeichen, welche hierbei für ein Entzündungsgeschehen sprechen, gehören u. a.:

- eine Unschärfe oder Unregelmäßigkeit der Aneurysmawand,
- ein periaortisches Ödem (sowie)
- eine Abszedierung im M. psoas [23].

Bei einer durch Blutkulturen bereits bestätigten Bakteriämie mit bisher erfolgloser Fokussuche kann im Sinne einer letzten Option das unter Fall 2 eingesetzte PET-CT angewandt werden [2].

Symptomatisch fällt das MBAA laut den in der Literatur beschriebenen Fällen am häufigsten durch das Vorhandensein von Schmerzen (45,0 %) sowie von Fieber (44,6 %) auf. Selten kam es noch vor der Diagnosestellung zum Schock (5,3 %). In 29,7 % der beschriebenen Fälle lag bereits eine Ruptur vor.

Am häufigsten traten MAAs im Bereich der infrarenalen Aorta abdominalis auf (35,1 %). Sehr selten kam es auch zu multiplen Lokalisationen (1,3 %).

Blutkulturen waren in 62,6 % positiv. Am häufigsten identifiziert wurden dabei Erreger der *Salmonella* spp. (26,3 %) sowie der *Staphylococcus* spp. (13,9 %) und der *Streptococcus *spp. (10,5 %). Zu beachten ist jedoch auch der nicht geringe Anteil an Patienten mit negativen Blutkulturen (37,4 %) bei histopathologisch bzw. mikrobiologisch nachgewiesenem MAA [3–22].

### Therapieoptionen

Als therapeutische Optionen kommen für die Behandlung des MBAAs grundsätzlich 2 Alternativen in Betracht: die OR mit Prothesenimplantation und das EVAR.

Die meisten Studien weisen gute Kurzzeitergebnisse für die Behandlung von MBAAs mittels EVAR auf [5, 6, 9, 11–13, 15–18]. Die 1‑Jahres-Überlebensrate lag bei 76–92 % für EVAR und 88,7 % für die OR [9, 15, 22]. Bezüglich der 3‑Monate-Überlebensrate schnitt EVAR mit 96 % sogar besser ab als die OR mit 74 % [16]. Nur wenige Studien enthalten Langzeitergebnisse. Die 5‑Jahres-Überlebensraten der verschiedenen Publikationen weisen eine Spannweite von 57–79,7 % für die Therapie mittels EVAR auf [4, 10]. Für die OR waren es dagegen konstante 60 % [15, 16].

Ebenfalls zu erwähnen ist, dass, obwohl Xenografts sowie autonomer Ersatz im Vergleich zu Allografts aus Kunststoff als deutlich infektresistenter gelten [15], in den meisten Quellen lediglich der alloplastische Ersatz beschrieben wird. Lediglich Berard et al. und Hassan et al. erwähnen auch Xenografts aus bovinem Material [4, 8]. Der autologe Ersatz kommt ebenfalls nur bei Berard et al. und Guo et al. zum Einsatz [4, 7].

Alle Studien außer einer (Skov et al., 2024) stellten eine häufigere Rate an Reinfektionen und infektionsbedingten Komplikationen nach EVAR als nach OR fest (24 % vs. 18 %) [15, 16].

Als besondere Risikofaktoren für das Auftreten eines MBAAs wurden ein mittleres Alter, männliches Geschlecht, vorbestehender Hypertonus, Diabetes mellitus und Atherosklerose festgestellt [7]. Als Prädiktoren für eine späte Mortalität wurden ein hohes Alter, das Vorhandensein von Symptomen wie Fieber und Schmerzen zum Zeitpunkt der Diagnosestellung, ein primär rupturiertes Aneurysma, eine suprarenale Lokalisation sowie eine nicht ausreichend lange Antibiotikatherapie von weniger als 6 Monaten festgestellt [7, 16].

### Fallspezifische Aspekte

Entsprechend der diskutierten Literaturempfehlungen – wie folgt:

- wenn die Patienten hämodynamisch stabil sind (oder präoperativ einen sanierbaren Abszess zeigen), sollte die OR eines MBAAs wegen des höheren perioperativen Risikos vorgenommen werden,
- für eher instabile Patienten sollte das EVAR aufgrund des besseren Kurzzeitüberlebens (und der häufigen Notwendigkeit von Re-Interventionen) Verwendung finden,

wurden auch die Entscheidungen in den oben beschriebenen Fällen getroffen: Im Fall von Patient 1 war präoperativ nicht bekannt, dass es sich um ein MBAA handelte. Zunächst wurde also die Entscheidung für eine OR des gedeckt rupturierten Aneurysmas getroffen und intraoperativ der Einsatz einer infektresistenteren Xenograft-Prothese beschlossen.

Bei Patient 3 fiel dagegen bereits präoperativ ein Psoasabszess auf, weshalb die Indikation zur OR mit Ausräumung des Abszesses und Implantation einer Xenograft-Prothese gerechtfertigt war.

Nach EVAR-assoziierten Richtlinien wurde beim beschriebenen Patienten 2 vorgegangen.

Die Dringlichkeit der bildgebenden Diagnostik in Fall 1 wurde zunächst durch die Vorbehandler unterschätzt, weshalb die finale Diagnose- und Indikationsstellung zur notfallmäßigen operativen Versorgung erst am Folgetag nach stationärer Aufnahme erfolgte.

## Fazit für die Praxis

- Das MBAA stellt ein seltenes Krankheitsbild bei erheblicher und damit beachtenswerter Mortalität dar. Hinweisend sind Fieber oder Sepsiszeichen, insbesondere wenn bei männlichen Patienten mittleren Alters ein BAA bereits vorbekannt ist.
- Als Therapie kommt entweder eine OR oder EVAR infrage. Da es sich bei den aktuellen Studien zu Therapiestrategien des MBAAs allerdings um retrospektive mono- oder multizentrische Studien handelt, enthalten diese oft keine Einzeldaten von Patienten. Es werden auch keine genaueren Informationen zum jeweiligen Patientenkollektiv und dem Prozess der Entscheidungsfindung gegeben, welche Patienten welche Therapie erhielten. Auch werden keine Informationen gegeben, ob zum Zeitpunkt der Therapieentscheidung bereits bekannt war, dass es sich um ein MBAA handelte oder nicht. Die einzige Angabe zur Therapieentscheidung während der Studien umfasste, dass die Entscheidung nach Präferenz des Operationsteams getroffen wurde.
- In Anbetracht der Studienlage sollte die OR eines MBAAs aufgrund des höheren perioperativen Risikos eher bei solchen Patienten zum Einsatz kommen, die hämodynamisch stabil sind oder präoperativ einen aussichtsreich sanierbaren Abszess aufweisen.
- Das EVAR sollte aufgrund des besseren Kurzzeitüberlebens und der häufigen Notwendigkeit von Re-Interventionen eher für instabile Patienten eingesetzt werden, welche die entsprechenden Bedingungen für eine EVAR erfüllen.
- Letztlich muss die Therapieentscheidung immer in Abhängigkeit von der Dringlichkeit, Rupturgefahr, hämodynamischen Stabilität und Multimorbidität der Patienten getroffen werden. Um Komplikationen durch Reinfektionen zuvorzukommen, sind außerdem regelmäßige Verlaufskontrollen zu empfehlen.
- Eine in allen Studien übereinstimmend vertretene Empfehlung ist hingegen die Notwendigkeit einer Langzeitantibiotikatherapie über mindestens 6 Wochen, idealerweise bis zu 6 Monaten.

## Data Availability

Die Daten stehen im Bedarfsfall zur Verfügung und werden vom Server des Universitätsklinikums Magdeburg A. ö. R. &/oder vom persönlichen Laptop des „senior authors“, Dr. Udo Barth, bereitgestellt.

## References

[1] Sorelius K, Mani K, Bjorck M, Sedivy P, Wahlgren CM, Taylor P et al (2014) Endovascular treatment of mycotic aortic aneurysms: a European multicenter study. Circulation 130(24):2136–2142 25378548 10.1161/CIRCULATIONAHA.114.009481

[2] Wilson WR, Bower TC, Creager MA, Amin-Hanjani S, O’Gara PT, Lockhart PB et al (2016) Vascular Graft Infections, Mycotic Aneurysms, and Endovascular Infections: A Scientific Statement From the American Heart Association. Circulation 134(20):e412–e460 27737955 10.1161/CIR.0000000000000457

[3] Barry IP (2019) Mycotic Abdominal Aortic Aneurysm in the Endovascular Era. Cureus 11(11):e6119 31886057 10.7759/cureus.6119 PMC6903872

[4] Berard X, Battut AS, Puges M, Carrer M, Stenson K, Cazanave C et al (2022) Fifteen-year, single-center experience with in situ reconstruction for infected native aortic aneurysms. J Vasc Surg 75(3):950–961 34600030 10.1016/j.jvs.2021.08.094

[5] Dang Q, Statius van Eps RG, Wever JJ, Veger HTC (2020) Dutch Society of Vascular Surgery, the Steering Committee of the Dutch Surgical Aneurysm Audit, and the Dutch Institute for Clinical Auditing. Nationwide study of the treatment of mycotic abdominal aortic aneurysms comparing open and endovascular repair in The Netherlands. J Vasc Surg 72(2):531–540 32061482 10.1016/j.jvs.2019.09.060

[6] Dimitrief M, Cherbanyk F, Deglise S, Pezzetta E (2016) Contained rupture of a mycotic infrarenal aortic aneurysm infected with Campylobacter fetus. BMJ Case Rep 2016:bcr2016215582. 10.1136/bcr-2016-215582 PMC5128960 27852656

[7] Guo Y, Bai Y, Yang C, Wang P, Gu L (2018) Mycotic aneurysm due to Salmonella species: clinical experiences and review of the literature. Braz J Med Biol Res 51(9):e6864 29947649 10.1590/1414-431X20186864 PMC6040868

[8] Hassan A, Khan A, Huasen B, Banihani M (2021) Aortoenteric fistula after endovascular mycotic aortic aneurysm exclusion: lessons learned during the COVID-19 era. BMJ Case Rep 14(2):e238875 10.1136/bcr-2020-238875 PMC10577721 33547124

[9] Jacobs CR, Scali ST, Khan T, Cadavid F, Staton KM, Feezor RJ et al (2022) Endovascular aneurysm repair conversion is an increasingly common indication for open abdominal aortic aneurysm repair. J Vasc Surg 75(1):144–152 (e1) 34314833 10.1016/j.jvs.2021.07.121

[10] Jutidamrongphan W, Kritpracha B, Sorelius K, Chichareon P, Chongsuvivatwong V, Sungsiri J et al (2023) Predicting Infection Related Complications After Endovascular Repair of Infective Native Aortic Aneurysms. Eur J Vasc Endovasc Surg 65(3):425–432 36336285 10.1016/j.ejvs.2022.11.003

[11] Lim YT, Tay WM, Lo ZJ, Pua U, Quek LHH, Tan BP et al (2022) Endovascular repair of mycotic aortic aneurysms confers good medium-term outcomes and aneurysmal sac resolution. Singap Med J 63(5):263–267 10.11622/smedj.2020165 PMC9297181 36043296

[12] Luo Y, Zhu J, Dai X, Fan H, Feng Z, Zhang Y et al (2018) Endovascular treatment of primary mycotic aortic aneurysms: a 7-year single-center experience. J Int Med Res 46(9):3903–3909 29962258 10.1177/0300060518781651 PMC6136017

[13] Orellana Davila B, Mancusi C, Coscarella C, Spataro C, Carfagna P, Ippoliti A et al (2024) Urgent or Emergent Endovascular Aortic Repair of Infective Aortitis. J Clin Med 13(16):4669 10.3390/jcm13164669 PMC11354867 39200812

[14] Scali ST, Waterman A, Feezor RJ, Martin TD, Hess PJ Jr., Huber TS et al (2013) Treatment of acute visceral aortic pathology with fenestrated/branched endovascular repair in high-surgical-risk patients. J Vasc Surg 58(1):56–65 23706619 10.1016/j.jvs.2012.12.043 PMC4183351

[15] Skov RAC, Lawaetz M, Eldrup N, Resch TA, Sorelius K (2024) Danish Academic Research Consortium for I. Danish Nationwide Study on Surgical Treatment of Infective Native Abdominal Aortic Aneurysms. Eur J Vasc Endovasc Surg 68(1):110–118 37944790 10.1016/j.ejvs.2023.11.006

[16] Sorelius K, Wanhainen A, Furebring M, Bjorck M, Gillgren P, Mani K et al (2016) Nationwide study of the treatment of mycotic abdominal aortic aneurysms comparing open and endovascular repair. Circulation 134(23):1822–1832 27799273 10.1161/CIRCULATIONAHA.116.024021

[17] Sorelius K, Budtz-Lilly J, Mani K, Wanhainen A (2019) Systematic Review of the Management of Mycotic Aortic Aneurysms. Eur J Vasc Endovasc Surg 58(3):426–435 31320247 10.1016/j.ejvs.2019.05.004

[18] Sule JA, Dharmaraj RB (2016) Surgeon Modified Fenestrated Endovascular Abdominal Aortic Repair (F-EVAR) for Subacute Multifocal Mycotic Abdominal and Iliac Artery Saccular Aneurysms. Ejves Short Rep 32:7–11 28856307 10.1016/j.ejvssr.2016.03.007 PMC5576004

[19] Sunil SP, Hanif H (2024) A 5-year retrospective study of abdominal aortic aneurysm repair in a Malaysian hospital. Med J Malaysia 79(1):42–46 38287756

[20] Tong TK, Shan G, Sibangun FJ, Keung BLD (2021) Melioidosis-related mycotic aneurysm: Three cases. IDCases 26:e1295 34646734 10.1016/j.idcr.2021.e01295 PMC8496099

[21] Touma J, Couture T, Davaine JM, de Boissieu P, Oubaya N, Michel C et al (2022) Mycotic/Infective Native Aortic Aneurysms: Results After Preferential Use of Open Surgery and Arterial Allografts. Eur J Vasc Endovasc Surg 63(3):475–483 34872811 10.1016/j.ejvs.2021.10.041

[22] Yi S, Sheng L, Li W (2022) Therapeutic effectiveness of tuberculous aneurysm and risk factors for mortality: a systematic review. Gen Thorac Cardiovasc Surg 70(6):515–525 35378674 10.1007/s11748-022-01811-9 PMC9135858

[23] Macedo TA, Stanson AW, Oderich GS, Johnson CM, Panneton JM, Tie ML (2004) Infected aortic aneurysms: imaging findings. Radiology 231(1):250–257 15068950 10.1148/radiol.2311021700

